# MIReAD, a minimum information standard for reporting arthropod abundance data

**DOI:** 10.1038/s41597-019-0042-5

**Published:** 2019-04-25

**Authors:** Samuel S. C. Rund, Kyle Braak, Lauren Cator, Kyle Copas, Scott J. Emrich, Gloria I. Giraldo-Calderón, Michael A. Johansson, Naveed Heydari, Donald Hobern, Sarah A. Kelly, Daniel Lawson, Cynthia Lord, Robert M. MacCallum, Dominique G. Roche, Sadie J. Ryan, Dmitry Schigel, Kurt Vandegrift, Matthew Watts, Jennifer M. Zaspel, Samraat Pawar

**Affiliations:** 1 VectorBase, Department of Biological Sciences, University of Notre Dame, Notre Dame, IN USA; 2 Universidad Icesi, Facultad de Ciencias Naturales, Calle 18 No. 122-135, Cali, Colombia; 3 Global Biodiversity Information Facility (GBIF) Secretariat, Copenhagen, Denmark; 4 Department of Life Sciences, Imperial College London, Silwood Park Campus, Buckhurst Road, Ascot, Berkshire, United Kingdom; 5 Department of Electrical Engineering and Computer Science, University of Tennessee, Knoxville, TN USA; 6 Division of Vector-Borne Diseases, Centers for Disease Control and Prevention, 1324 Calle Cañada, San Juan, PR USA; 7 Department of Epidemiology, Harvard School of Public Health, 677 Huntington Ave, Boston, MA USA; 8 Center for Global Health and Translational Science, State University of New York Upstate Medical University, Syracuse, NY USA; 9 VectorBase and Vector Immunogenomics and Infection Laboratory, Department of Life Sciences, Imperial College London, London, United Kingdom; 10 Florida Medical Entomology Lab, University of Florida-IFAS, Vero Beach, FL USA; 11 Institute of Biology, University of Neuchâtel, 2000 Neuchâtel, Switzerland; 12 Quantitative Disease Ecology and Conservation Lab, Department of Geography, University of Florida, Gainesville, FL USA; 13 Emerging Pathogens Institute, University of Florida, Gainesville, FL USA; 14 College of Life Sciences, University of Kwa-Zulu Natal, Durban, South Africa; 15 Center for Infectious Disease Dynamics, Department of Biology, The Pennsylvania State University, University Park, PA USA; 16 Department of Zoology, Milwaukee Public Museum, Milwaukee, WI USA

**Keywords:** Population dynamics, Ecological epidemiology, Research data

## Abstract

Arthropods play a dominant role in natural and human-modified terrestrial ecosystem dynamics. Spatially-explicit arthropod population time-series data are crucial for statistical or mathematical models of these dynamics and assessment of their veterinary, medical, agricultural, and ecological impacts. Such data have been collected world-wide for over a century, but remain scattered and largely inaccessible. In particular, with the ever-present and growing threat of arthropod pests and vectors of infectious diseases, there are numerous historical and ongoing surveillance efforts, but the data are not reported in consistent formats and typically lack sufficient metadata to make reuse and re-analysis possible. Here, we present the first-ever minimum information standard for arthropod abundance, Minimum Information for Reusable Arthropod Abundance Data (MIReAD). Developed with broad stakeholder collaboration, it balances sufficiency for reuse with the practicality of preparing the data for submission. It is designed to optimize data (re)usability from the “FAIR,” (Findable, Accessible, Interoperable, and Reusable) principles of public data archiving (PDA). This standard will facilitate data unification across research initiatives and communities dedicated to surveillance for detection and control of vector-borne diseases and pests.

## Introduction

Arthropods play a dominant role in the dynamics of practically all natural and human-modified terrestrial ecosystems^1–3^ and have significant economic and health effects. For example, certain insects provide significant economic benefits (*e*.*g*. pollination) exceeding $57 billion a year to the United States alone^4^. Invasive insects, however, cost an estimated $70 billion dollars per year globally^5^ and insect pests may reduce agricultural harvests by up to 16%, with an equal amount of further losses of harvested goods^6^. Particularly noteworthy is a subset of arthropods that are disease vectors, transmitting pathogens to and between animals as well as plants. Vector borne diseases cause billions of dollars in crop and livestock losses, every year^7–9^. In humans, vector borne diseases account for more than 17% of all infectious diseases (*e*.*g*. malaria, Chagas, dengue, and leishmaniasis, Zika, West Nile, Lyme disease, and sleeping sickness), with hundreds of thousands of deaths, hundreds of millions of cases, and billions of people at risk, annually^10,11^.

**Fig. 1:**
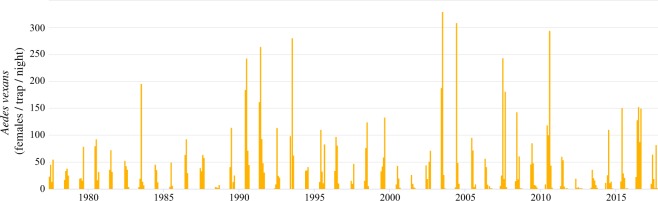
Population abundance time-series example. From New Jersey light trap mosquito surveillance performed by the Iowa State University Medical Entomology Laboratory from 1977-2017^61,62^. Data available for download on VectorBase^50^.

The current economic and health burden of arthropod pests, exacerbated by invasive species, and uncertain effects of climate change^12,13^, has driven significant research programs and data collection efforts. These include crop pest, mosquito, and tick survey and reporting initiatives^14–18^, citizen science projects^19–21^, and digitization of museum specimen data^22,23^, all yielding a rich and growing trove of field-based data spanning multiple spatial and temporal scales. The monitoring of arthropod abundance (*e*.*g*. Fig. 1) in different disciplines (*e*.*g*., biodiversity research, pest-control assessment, vector borne disease monitoring, and pollination research) has similar objectives — to quantify abundance, phenology and geographical ranges of target arthropod species — and entails similar techniques. However, the data produced by these various efforts are often not reusable, or comparable to similar data, as they are typically not recorded in a standard format (*e*.*g*. Darwin Core), or do not provide adequate metadata. In contrast, the advent of journal-mandated deposition of data from high-throughput technologies (*e*.*g*. NCBI and GenBank), data and code sharing, and other practices to improve transparency and reusability of research results are increasing rapidly across the sciences^24–29^. Furthering these advances through standardization and public archiving of arthropod abundance data can bring significant benefits, including (1) supporting empirical parameterization and validation of mathematical models (*e*.*g*. of pest or disease emergence and spread), (2) validation of model predictions, (3) reduction in the duplication of expensive empirical research, and (4) revealing new patterns and questions through meta-analyses^11,30–33^. This will also lead to substantial public benefit through improved human, animal, plant, and ecosystem health, and reduced economic costs.

One of the key impediments to the re-use of these data is the lack of adequate metadata or data descriptors (*i*.*e*. data about the data)^34–37^. In general, for data to be most valuable to the scientific community, they should meet the FAIR Principles – they should be Findable, Accessible, Interoperable and Reusable – and delineate the key components of good data management and stewardship practices^38,39^. Data are Findable and Accessible when they are archived and freely downloadable from an online public data repository that is indexed and easily searchable. Interoperability and reusability describe the ease with which humans or computer programs can understand the data (*e*.*g*. via metadata) and explore/re-use them across a variety of non-proprietary platforms. Even when data are available, metadata for arthropod abundance data are often absent or not readily interpretable, limiting their reusability at a fundamental level.

## Results

### A minimum information standard for arthropod abundance data

Here, we present a Minimum Information for Reusable Arthropod Abundance Data (MIReAD) standard for reporting primarily longitudinal (repeated, temporally explicit) field-based collections of arthropods. ‘MIReAD’ also evokes ‘Myriad,’ a countless or extremely great number. Abundance is measured and reported in different ways, and MIReAD fields have been designed to allow researchers to capture this complexity. Examples 1–4 (which can be found in Figshare^40^), provide examples for how to report such different types of abundances. However, we do not encourage the reporting of relative population abundances since these are not raw data as such, but derived values. One might argue that this could lead to (and it probably will) loss of information, but we argue that the reporting of raw abundance or occurrence data is non-negotiable if these data are to be reused. For example, incorrect statistical methods to aggregate data, such as taking the arithmetic mean for skewed abundance data across samples or replicates are not uncommon, and we wish to discourage such practices. In the same manner as has been developed in other biological disciplines^41–46^, this standard is “minimum” because it defines the necessary minimal information required to understand and reuse a dataset without consulting any further persons, text, materials, or methods^47^. MIReAD is designed to facilitate data archiving efforts of publishers and field researchers. It is not a data model [the explicit definition of data field names, data formats (*e*.*g*., for dates and GPS locations)] and therefore does not define controlled vocabularies, or specific field titles, but should be easy to understand and interpret by the wider scientific community^47^.

**Fig. 2:**
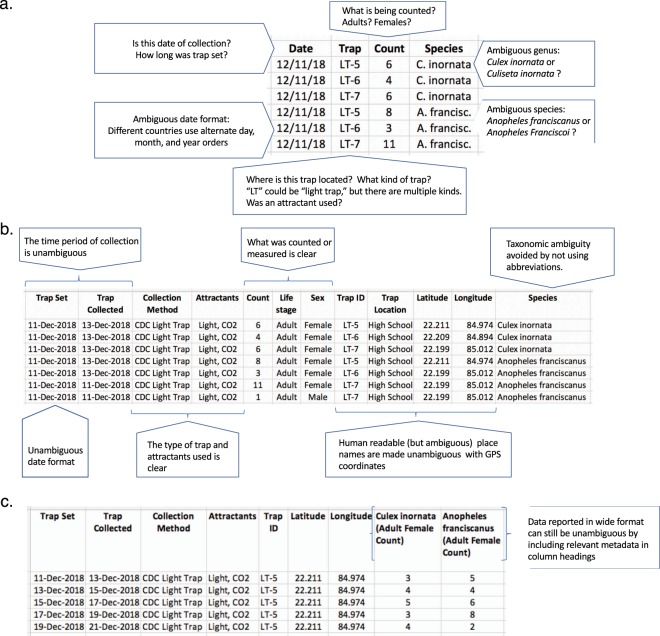
MIReAD reduces data ambiguity. (**a**) Seemingly clean data can still lack key information or have ambiguous metadata, hindering data reuse. (**b**) MIReAD compliant data includes the metadata necessary for data reuse and removes ambiguity. (**c**) Note data can be formatted differently, but still be MIReAD compliant such as by presenting data in a wide format.

MIReAD is separated into two components, metadata and data. For each component, we provide a description of the information that should be included, recommendations for how to make that information as useful as possible, and examples. The metadata component (Online-only Table 1) includes information for the origin of the data set (*e*.*g*. study information and licensing for usage). The second component (Online-only Table 2) lists and describes specific data fields that should be included in data collection sheets. We also provide recommendations and examples to demonstrate how these recommendations can be implemented. MIReAD was designed to match the data that are generally collected by academic researchers and surveillance initiatives, and can serve as a checklist for important information that needs to be recorded but is often unintentionally omitted (*e*.*g*. Fig. 2a). By adhering to MIReAD standards, omissions and ambiguity can be avoided even if the data are shared in different formats (Fig. 2b,c). Finally, we identify common problems likely to be encountered across all the MIReAD metadata and data fields, and data quality standards that can be employed to avoid confusion (Box 1).

Box 1 Data quality standards **No abbreviations**. Abbreviations (including in columns names) are ambiguous, with the exception of measurement units (*e*.*g*. centigrade and meters).

**No external legend/key files**. While repetitive, all data should be explicitly given within the data table. Separate files mapping ID numbers to GPS locations, full species names, *etc.* should be avoided. In addition, rich metadata are essential for good data discovery and reuse.

**Unambiguous dates**. Because of country-level differences in date formats, data should be reported unambiguously with four-digit years, and months provided alphabetically and not numerically (*e*.*g*. 4-Jun-2017 or Nov 12, 2015,) or by using ISO 8601 date format (YYYY-MM-DD, *e*.*g*. 2019-01-27)

**Machine-readable file formats**. Data should be provided in non-proprietary machine-readable formats such as comma-separated text files. PDFs and multiple spreadsheets in the same document should be avoided.

**No font styling or subsection headings**. Formatting (color, bold, italics, subscripts, sheet tab names, *etc*.) should not be required for understanding the data. Subsection headings should not be required to understand data; every line of data should be interpretable in isolation from any other line of data.

**Highest precision possible**. Data should be provided at the highest temporal, spatial, numerical, and taxonomic resolution available. If location (*e*.*g*., geographical coordinate) data need to be presented at a lower resolution than available for privacy reasons, this should be made clear in the submission in Study Information (Resource Metadata; Online-only Table 1).

**Language**. Once data are ready to be deposited/submitted, all fields and data should be preferably written in English. This will allow researchers and data curators worldwide to understand and reuse the data. Use of other languages is better than not publishing data. Please avoid introducing data reuse barriers through incomplete translation. For example, avoid non-English field names in an English-language submission.

### Examples

Below we provide three examples to illustrate MIReAD-compliant data (linked to examples 1–4 in Figshare^40^, respectively). Researchers can use these data sheets as a basis for formatting their own data. In these examples, note that all data meet the data quality standards of Box 1; are adequately described, have columns labeled, *etc*. to eliminate ambiguity (even if the data appear repetitive; for example, the sex and life stage are repeated in every row). Examples 1 and 2 should be sufficient for most data generators. Examples 3–4 demonstrate more complex data collection scenarios.

#### Long-format trapping data

Each row captures count data for a single species’ occurrence in a given sampling event. This illustrates an example of the most common mosquito collection protocol (MIReAD_example_1.csv^40^). Also see Fig. 2b.

#### Wide format trapping data

Each row captures count data from a given sampling event. Each identified taxonomic group is identified in a separate column. An ‘additional sample information’ field, ‘sub-location,’ has been added to describe the various locations around the village where collections were made (MIReAD_example_2.csv^40^). This illustrates an example of adult mosquito populations that have been tracked over time and in specific locations. Also see Fig. 2c.

#### Complex trapping data scenarios

Tick surveillance performed using tick drags and flags and collections of ectoparasites on trapped mice. The tick drags/flags report three life stages independently (adult, larvae, and nymph) (MIReAD_example_3.csv^40^). Larvae are only identified to the genus, while adults and nymphs are identified to the species. Observations of different life stages and sexes are preferably documented in separate records. A Sample Name is used to help link these records (but would not be necessary.) The mouse survey uses an additional sample information field to record the sex of the trapped mouse from which the parasites were collected (MIReAD_example_4.csv^40^).

## Discussion

### MIReAD as the path to FAIR data principles

We designed MIReAD to achieve a balance between standards that are too onerous for data generators with guidelines that are sufficient to ensure at least minimal reusability^31,41^. It balances a perfectly formatted and reusable dataset with all necessary metadata in a consistent format (but comes with a high burden on the dataset generator) versus a dataset that is unusable or re-usable due to missing or incomplete metadata. MIReAD allows for a relatively easy standardization, as it ensures all necessary collection metadata is present in an unambiguous manner. By not mandating any particular field name, field order, or controlled vocabulary terms, we will in fact gain traction from other more rigorous (and thus more onerous) data models, for which lack of minimum standards in data are often a first and major hurdle. In striking this balance, we note that MIReAD focuses on capturing information on ‘what’ was done, rather than ‘why’. We acknowledge that for some use cases, this may hinder reusability^48^ but for the majority of cases, where the results of the original data can be interpreted without understanding the rationale, providing data in MIReAD format will be sufficient for data reuse.

Like all minimum standards, MIReAD only aims at ensuring data ‘Reusability’. However, ultimately this will promote the implementation of data models, and controlled vocabularies (*e*.*g*., the Darwin Core^49^). Data models enable ‘Interoperability’, and in turn facilitate structured databases, public repositories, and development of data analysis tools^47,50^. Deposition in open databases make data ‘Findable’ and ‘Accessible’^51–53^. MIReAD compliant data contain sufficient information for established aggregators/databases such as VectorBase and SCAN (Symbiota Collections of Arthropods Network^54^) to process and store the data in a standardized data model [*e*.*g*., Darwin Core, a widely used universal data standard that supports opportunistic observation and collection data (occurrence core) as well as presence/absence and abundance data collected using strict and documented methodology (event core)^49^], and ultimately facilitate data transfer to even more comprehensive biodiversity databases. For example, GBIF contains over one billion species occurrence records, from thousands of environmental, ecological, and natural resource investigations, including research on Arthropoda in numerous ecological and monitoring projects, allowing for study of changes and trends in populations^53^. Indeed, in Tables 1, 2, we provide an example of the mapping of data fields from MIReAD to DarwinCore and GBIF. In this way, MIReAD opens the door to FAIR data and more sophisticated methods to integrate data across many scales.

**Table 1:** The MIReAD data fields and their mapping to the GBIF metadata profile.

MIReAD Field	Corresponding GBIF metadata fields
Contact details	contact
General description of the experiment/collection set	designDescription; sampling
Citations	citation
Species Identification Method	designDescription
Not present vs zero information	samplingDescription
GPS obfuscation information	geographicDescription geodeticDatum
Data usage information	intellectualRights

See GBIF^63^ for more information.

**Table 2:** The MIReAD data fields and their mapping to the Darwin Core data standard.

MIReAD Field	Corresponding DarwinCore fields
Start Time (for collection)	eventTime
End Time (for collection)	eventTime
Location	A number of fields under Location See: http://rs.tdwg.org/dwc/terms/#location
Collection method	samplingProtocol
Collection attractants	samplingProtocol
Collection area	samplingEffort
Taxonomy	A number of fields under Taxon See http://rs.tdwg.org/dwc/terms/#taxon
Unit(s) of measurement and observation	sampleSizeUnite
Value	sampleSizeValue
Additional sample information	fieldNotes
	eventRemarks
SampleID	eventID
Sample Name	*e*.*g*. fieldNumber for individual observations; see also SampleID above for the field names for the complex samples

See Wieczorek *et al*.^64^ for more information.

### Benefits to field researchers

It is essential that the benefits of a minimal data standard extend not just to data re-users, but also to the researchers who collect and generate data in the first place. MIReAD provides a framework for data preparation that can help scientists achieve recognized professional merit for sharing data such as increased citation rates, academic recognition, opportunities for co-authorship, and new collaborations [sensu Roche *et al*.^31^]. Large, deposited data sets can now themselves be standalone, citable “data papers” (*e*.*g*.^55–57^) or even depositions without any traditional manuscript (but as an authored ‘digital product,’ with persistent identifiers, such as a DOI number), if desired. Data sets are increasingly recognized as valuable research outputs that count towards academic recognition and professional advancement (*e*.*g*. grants, interviews, and tenure). For example, several funders (*e*.*g*. United States National Science Foundation and Swiss National Science Foundation) have adopted or are in the process of adopting the Declaration on Research Assessments (DORA)^58^, offering further opportunities for data generators to gain recognition and publication credit for their work^59^. Also, an increasing number of funders are mandating public data access, and detailed data management plans are often required even at the grant proposal stage. Therefore, reporting data according to MIReAD will provide a basis for stipulating archival formats. We also note that by storing data in MIReAD format, data generators can assure that their data contains all the necessary metadata for their own internal use. As time passes, research staff, sampling protocols, and sampling locations change, and thus the recording of minimal information ensures long-term reusability of data.

Furthermore, many data generators are also data users. Developing analyses that rely on standardized fields can facilitate the development of generalized analytical tools that can be easily extended to datasets beyond those that were collected by a single individual or lab. In this way, they can enable extensions of work that would otherwise not happen, such as comparisons of population dynamics in different locations or assessments of interspecies interactions. Adopting MIReAD can, therefore, both help data generators reap the benefits of sharing data they have collected and enable them to more readily leverage data collected by others.

### Further MIReAD applications and extensions

The creation of minimum information standards for these types of databases facilitates analyses of data at scales that cannot be attained by a single individual or lab group. Linking records to additional information also extends the utility of these data to address population level questions. For example, a well-populated database presents opportunities to investigate interactions between populations of different species of arthropod that overlap in geography but may be of interest individually to different realms of research. As a case in point, in the northeastern USA, *Agrilus plannipennis*, the Emerald Ash Borer, is a highly destructive invasive insect, monitored closely by both state and federal agencies for management^60^. Interestingly, Emerald Ash Borers are creating new habitats for carpenter bees, a species interaction that can be tracked and anticipated using large scale arthropod data.

Another example of the utility of linked data is for disease vectors. Data on insecticide resistance linked with time and place would be valuable for coordinating control strategies within and between nations and communities. Presence/absence data on infection levels would be helpful for tracking and investigating disease outbreaks and dynamics. Standardization of these data would be particularly useful for pathogens that infect multiple vectors and hosts and would facilitate a “One Health” approach. Other important vector phenotypes that contribute to control and transmission such as pathogen susceptibility, biting preferences, and breeding behaviours could be measured over time and space.

Indeed, MIReAD would be useable for any arthropod abundance data collection effort, not just medical, veterinary, and agricultural pests or  invasive arthropods. We note this standard is applicable not only to abundance measurements, but could be easily extended to any other kind of routinely sampled time-series field data. For example, in addition to aphid abundance, plant pathogen (such as mosaic virus) infection and insecticide resistance statuses of the aphids could be reported in MIReAD format. Note that MIReAD can also be used for cross-sectional data (*i*.*e*. *a* non-continuous, one-time sampling effort) by simply reporting data from the single collection period by utilizing a single Start Time and End Time (Online-only Table 2).

### Disseminating MIReAD

Many data generators are already storing or sharing data in a manner that would be consistent with MIReAD (*e*.*g*. on MosquitoNet or NEON), but we call on data generators, authors, reviewers, editors, journals, research infrastructures (*e*.*g*. data repositories) and funders to embrace MIReAD as a standard to facilitate FAIR data use and compliance for arthropod abundance data. We propose that workshops, outreach at conferences and meetings, and interfacing with data repositories, societies and organizations (*e*.*g*. SpeciesLink, the American Mosquito Control Association, MosquitoNet, Symbiota, VectorNet, and VectorBiTE), and journal editors will be the best way to spread the adoption of this standard.

## Conclusion

We present MIReAD as a minimum information standard for representing arthropod abundance data. MIReAD will facilitate collation and analyses of data at scales that cannot be attained by a single individual or lab in order to address key questions across temporal and spatial scales, such as within and across-year phenology of abundance of target arthropod taxa over large geographical areas. This is particularly important given the pressing need to understand and predict the population dynamics of harmful (*e*.*g*., disease vectors and pests) as well as beneficial (*e*.*g*., pollinators, bio-control agents) arthropods in natural and human modified landscapes. This is the first step for achieving the broad benefits of FAIR data for arthropod abundance.

## Data Availability

No novel data were generated for this report. We encourage readers to view the datasets that inspired and informed our work at www.vectorbase.org, www.gbif.org, www.vectorbyte.org, and in our other publication^14^.

## Online-only Table

**Online-only Table 1:** The MIReAD Study Information (Resource metadata) fields. The information in this table should be included with every data submission, for example by including data in the file header as demonstrated in the examples^40^

Field	Details	Recommendations	Examples
Contact details	A name, person, authority, *etc*. that may be contacted with enquiries about the data.	Include investigator ORCID(s), email address, website (if institutional) if possible.	Kurt Vandegrift orcid.org/0000-0002-5690-3300 kurtvandegrift@gmail.com State University Agricultural Extension John Smith (jsmith@StateU.edu) www.StateU.edu/AgriculturalExtension/
General description of the experiment/ collection set	A short description of the study objectives, sampling design, and hypotheses. Used to aid in browsing multiple studies. A short title and long form name might be helpful.	Useful things to indicate are: Random sampling or continuous monitoring in fixed locations General time frames and location. General description of where data is from. Subsampling details, if relevant. Rationale for trap placement, experimental design, *etc.* may be provided, if relevant.	“Monitoring of major pests on cucumber, sweet pepper and tomato under net-house conditions in Punjab, India” “Pennsylvania *Ixodes scapularis* weekly abundance” Continuous (weekly) monitoring of tick numbers attached to white-footed mice in fixed locations in Pennsylvania, USA (12 sites). 2003-present.” “Long term aphid emergence monitoring using continuous suction traps”
Citations	Reference to related publications, digital if possible (*e.g.* DOI(s) or PMID(s)).		“A web-based relational database for monitoring and analyzing mosquito population dynamics Sucaet Y, Van Hemert J, Tucker B, Bartholomay L.” “PMID: 18714883” “Horiuchi, Kaho, Kosei Hashimoto, and Fumio Hayashi. Cantharidin world in air: Spatiotemporal distributions of flying canthariphilous insects in the forest interior. Entomological Science (2018).”
Species Identification Method	A description of method of species identification. Particularly important for cryptic species complexes.	Providing information on the veracity of the identification is encouraged, such as a reference to the exact identification key or method.	“Morphological” “Genotyped, using method of Smith *et al*. 2014, PMID: 18714883” “Morphological: used keys of Doe (1958)” “High confidence morphological ID by Jane Doe”
Not present vs zero information	Indication of what gaps, zeros, NA, *etc.* mean.	It is imperative, especially for population surveys, to understand the difference between a species was not found when the collection method would be expected to find the given species (confirmed absence), or a species was not looked for (*e*.*g*. a trap failure) Preferably, a zero indicates a species was looked for and not found, and a NA represents a species was not looked for/trap failure/ etc. Blank values are highly discouraged Authors might also assert a definition of absence.	“Zero indicates a species was looked for and not found. NA represents a trap failure” “When a species is indicated as absent, it was expected due to extensive sampling and found in other places during the study but not found here.”
GPS information	If raw GPS data is obfuscated in any way, a statement on the manner by which this occurred should be given. The ellipsoid, geodetic datum, or spatial reference system (SRS), if known, can also be identified. GPS unit accuracy could also be provided.	The highest resolution data (*e*.*g*. trap-level, specific GPS location) are the most useful. It is hoped that no data obfuscation occurs. Some common GPS point obsfucations, if utilized, that should be noted: aggregation (making the areal unit larger, *e*.*g*. increasing pixel size in a raster (and generating new centroid coordinates) reducing precision of location (reducing GPS decimal points) dithering (‘moving’ GPS points via adding a degree of ‘error’ – distance – to the X,Y)	“Raw GPS points have been provided. GPS unit accuracy + /− 8 m” “GPS locations have had precision reduced by truncating to 3 decimals” “Points were dithered by displacing points randomly by X distance” “WGS84 datum used and data was aggregated to 1 km pixels”
Data usage information	The data reuse policy for your data. Please provide a creative commons license identification. See https://creativecommons.org for more information.	For data to be F.A.I.R., it must be Reusable. We therefore recommend data be provided as “CC0” or “CC BY 4.0”. “CC0”, under which data are made available for any use without restriction or particular requirements on the part of users “CC BY 4.0”, under which data are made available for any use provided that attribution is appropriately given for the sources of data used, in the manner specified by the owner (*e*.*g*. citation).	“CC 0” or “CC BY 4.0”

**Online-only Table 2:** The MIReAD data fields

Field(s)	Details	Recommendations	Examples
**Start Time (for collection)**	Start time of the data sample collection. *e*.*g*. The trap was set…	Be as specific as *practically* possible. Any unambiguous format is acceptable. However, do not use two-digit year abbreviations. If relevant, provide time zone in field or in header; a 24 hour clock is preferred but should be made unambiguous as to which time format is being used.	“2012-04-27” “July 26, 2017” “2017-Jul-26” “2017-July-26 Morning” “2017-Jul-26 20:00 GMT”
**End Time (for collection)**	End time of the data sample collection. *e*.*g*. The trap was collected…	See above. If instantaneous data collection (*e*.*g*. a tick drag), End Time may be the same as Start Time.	See above.
**Location**	The geographical location of sample collection.	As detailed as possible. Latitude and longitude if possible with specified accuracy Providing *both* a GPS point (decimalized GPS points are preferred) field and a geographical name field is preferred. Note only providing location *names* is highly discouraged as they change over time and can be ambiguous. Both Place / Trap names and GPS fields can be provided. If obfuscation was used, it should be indicated in the Metadata (Online-only Table 1). Splitting latitude and longitude further into two columns further reduces ambiguity.	“Kukar Maikiya, Jigawa State, Nigeria” and “40.697” and “ −74.015” “40.697” and “ −74.015”
**Collection method**	Sampling apparatus (*e*.*g*. trap type, observation method)		“CDC light trap” “Tick drag” “Quadrat count” “BG Sentinel Trap” “Pitfall trap” “Sticky trap” “Larval dip” “Johnson suction trap” “Lindgren Funnel Trap”
**Collection attractants**	The attractant/ lures used to attract insects to a trap or collection	Please be as specific as possible. For example, a cow used as bait should be referred to as a ‘cow’ and not ‘animal’, or specify the attractant used, such as ‘CO_2_’ Company names, in particular for chemical attractants, are helpful. Please explicitly state if no attractant was used.	“None” “Carbon dioxide” “UV light” “Biogents Sweetscent Mosquito Lure” “Human” "Russel IPM: CAT-QLURE-CB"
Collection area	The spatial extent (area or volume) of the sample.	If relevant (*e*.*g*., when collection method is transect or quadrat), in units of area or volume, the spatial coverage of the sampling unit. Note this field would not typically be used for passive collections from fixed traps.	“100 m^2” “1 liter” “1 ha” “10 m^3”
**Taxonomy**	Classification of sample collected.	Scientific genus and species preferred. Avoid abbreviation.	“*Chortoicetes terminifera*” “*Aedes aegypti*” “*Anopheles gambiae sensu stricto*” “*Chrysodeixis argentifera*“
**Unit(s) of measurement and observation**	Description of exactly what was observed, the unit for the field “Value,” below. For counts, should indicate life stage, sex, etc. Unit measures can be encoded into value field header. Consider multiple unit fields (*e.g.* separate fields for sex and stage.) See Fig. 2.	Do not abbreviate. Coded data key should be provided in field name (*e*.*g*. “1 = species present 0 = species absent”)	“Number of individuals per m^2” “Adult Females” “Males and Females” and “Nymphs”
**Value**	The numerical amount or result from the sample collection. Often this will be a quantity of observed individuals. Unit measures can be encoded into value field header. See Fig. 2.	Units should be provided in a separate field or in the header.	“0” “23” “Yes” “Not present”
Additional sample information field(s)	This could be more than one field and should be used when more information is required to understand the experiment, for example experimental variables, sub-locations, plant host cultivar/species, etc. Some users may report wind speeds, temperatures, elevations etc. This could also include data on disposition of any voucher specimen.	Do not abbreviate. For voucher specimens, it should be made known that a specimen is in fact a voucher and second, if different pieces of the voucher are stored separately (*e*.*g*., insect on pin, leg in freezer) or at different institutions.	“Forest” vs “Field” “Winter” vs “Summer” “Inside” vs “Outside” “200 meters above sea level” “Cowrie” vs “Leichhardt” vs “Surf” (Soybean cultivars)
Sample Name	A human readable sample name. May exist solely for the benefit of the depositor in organizing their data, use their own internal naming conventions etc. May also be used to tie related observations together. This field may be useful for updating records once deposited in a repository.	Naming convention is not restricted, but any encoded metadata should be revealed in the other data fields. For example, you may name a sample named ‘Aphid1_StickyTrap_Jan4,’ but you will still have “Sticky Trap” listed in a Collection Method field, and “Jan 4, 2017” in the date field.	“Trap1_Night1” “Armyworm_2” “00004” “Jan08_animal_4,” “ABC_123_4b”

Figure 2b provides an annotated example. Field names in bold should be considered required. Remaining fields are optional or depend on the complexity of the experimental design.
